# Antilisterial efficacy of *Lactobacillus brevis* MF179529 from cow: an in vivo evidence

**DOI:** 10.1186/s12906-019-2444-5

**Published:** 2019-02-01

**Authors:** Ayesha Riaz, Saleha Noureen, Iram Liqat, Muhammad Arshad, Najma Arshad

**Affiliations:** 1 Department of Zoology, University of the Punjab , Quaid-i-Azam Campus, Lahore, Pakistan; 2 Department of Zoology, Government College University, Lahore, Pakistan; 3 University of the Education, Lower Mall Campus, Lahore, Pakistan

**Keywords:** Prophylactic, *Listeria monocytogenes*, *Lactobacillus brevis*, Probiotic, Lactic acid bacteria

## Abstract

**Background:**

*Listeria monocytogenes* is an opportunistic foodborne pathogen that causes human Listeriosis and high mortality particularly in immunocompromised individuals. Pregnant women are more prone to *L. monocytogenes* infection resulting in abortions.

In the present study, antilisterial activity of *Lactobacillus brevis* (LB) MF179529, a probiotic bacterial strain, was investigated in a murine model.

**Methods:**

Initially a pilot study was conducted to determine the dose of *L. monocytogenes* required to cause symptomatic listeriosis. In the main trial, mice were divided into 4 groups. Group I was kept as negative control, group II was exposed to *L. monocytogenes* and maintained as positive control. Group III was fed with *L. brevis* only, while group IV received *L. brevis* for 3 days prior to *L. monocytogenes* infection. A volume of 200 μl of *L. monocytogenes* ATCC 19115 and *L. brevis* MF179529 bacterial suspension corresponding to cell density of 10^9^CFU/ml were given to respective groups by intragastric route. Progress of infection was monitored for 7 days including general health scoring, listeria dispersion in organs, bacterial load in intestine and blood biochemistry were recorded on 3rd, 5th and 7th days post infection (dpi).

**Results:**

Clinical listeriosis was induced by 10^9^CFU/ml of *L. monocytogenes* ATCC 19115 in mice.

Animals of group IV displayed minor signs of infection. *L. brevis* supplementation resulted in significant reduction in dispersion and propagation of *L. monocytogenes* in liver, spleen and intestine. *L. brevis* MF179529 consumption led to a significant elevation of number of lactic acid bacteria and reduction of total plate count, anaerobic count and coliform population in intestine. Moreover, total leukocyte and neutrophil counts of treated animals were similar to the negative control while positive control group displayed higher number. Safety evaluation of *L. brevis* was performed by monitoring general health, hematological and serological parameters of *L. brevis* fed and negative control group (group III and I). No significant difference in feed intake, body temperature, body weight and blood picture could be detected in *L. brevis* supplemented and control groups.

**Conclusion:**

Our results indicate ameliorative role of *L. brevis* in *L. monocytogenes* infection and suggest that *L. brevis* could be used for prophylactic measure.

**Electronic supplementary material:**

The online version of this article (10.1186/s12906-019-2444-5) contains supplementary material, which is available to authorized users.

## Background

Pathogenic microbes are important due to their role in health complications and food spoilage. *Listeria monocytogenes* (LM) is a facultative intracellular food borne pathogen which is causative agent of human listeriosis, gastrointritous, abortion, meningoencephalits and septicemia [1]. Immuno-compromised individuals, pregnant women and fetus are more prone to listeriosis [2]. Ingestion of LM contaminated food results in translocation of pathogen from intestinal lumen to different organs [3]. Listeriosis is serious public health issue and lead to 20 to 30% mortality [4].

Probiotics are defined as live nonpathogenic microorgansims, when used in adequate amount give health benefits to the host [5]. They play an important role in maintaining the microbial balance in gastrointestinal tract of the host by promoting growth of beneficial microbes and also modulate immune responses [6]. Mostly probiotics belong to a group of Lactic acid bacteria (LAB), among them *Lactobacillus* and *Bifidobacterium* genera are used commercially [7, 8]. Probiotic strains use different mechanisms to combat infection viz., production of inhibitory substances, blocking of adhesion sites on the intestinal surface, dislocation of the initially adhered pathogen, competition for nutrients and stimulation of mucosal and systemic immunity [9]. The antagonistic and co-aggregation potential of probiotic strains against pathogen is strain specific [10]. Probiotic supplements are used singly or in combination to combat pathogen but mixture of probiotic strains is reported to be more effective [11]. Probiotics are recommended by health professionals to cure gastroenteritis including mild diarrhea, ulcerative colitis, antibiotic associated diarrhea when standard theropies did not work [12].

Can be used as an alternative to chemical growth promoter, and growth performance of the host. LAB are reported to produce antimicrobial compound such as lactic acid, hydrogen peroxide, reuterin, diacetyl, acetoin reutricyclin, antifungal peptides and bacteriocins [13]. Bacteriocins are small peptides produced as secondary metabolites which have the ability to inhibit the growth of closely related species, allowing competition among different niches**.** LAB are known to produce fermented products as well as bacteriocins that have the inhibitory effect against clinical and foodborne pathogens including LM [14, 15]*.* Thus LAB are recommended to be used as food supplement and therapeutic agent against infectious diseases [16].

To the best of our knowledge, this is the first report on antimicrobial efficacy of *L. brevis* (LB) MF179529 against LM using a murine infection model**.**

## Methods

### Bacterial strain & culture conditions

*L. brevis* (LB) MF179529, a strain previously isolated from fresh cow fecal samples and identified by 16S rRNA sequencing in our laboratory. The bacterial strain was refreshed by plating on de Man Rogosa Sharpe (MRS) agar following incubation at 35 °C for 48 h anaerobically. This strain can be obtained from First Fungal Culture Bank of Pakistan (FCBP) with accession number FCBP-692, a registered World Federation of Culture Collections WFCC) in Pakistan. *Listeria monocytogenes* (LM) ATCC 19115 was obtained from the American Type Culture Collection. LM ATCC 19115 strain was cultivated in Tryptic Soya Broth (TSB) supplemented with 0.6% (*w*/*v*) yeast extract at 37 °C for 24 h. Both strains were preserved in 20% glycerol stocks at − 80 °C and were revived by sub culturing from glycerol stock whenever required.

### Preparation of inoculum

LB and LM were grown in de Man Rogosa Sharpe (MRS) and Tryptic Soya Broth (TSB) respectively for specified time period. In order to select the infectious bacterial density, overnight culture of LM was centrifuged and pellet was washed thrice with 0.01 M sterile Phosphate Buffered Saline (PBS) buffer (pH 7). Pellet was re-suspended in sterile 0.01 M PBS buffer, OD was adjusted to 1 ± 0.05 at 600 nm. Three doses were used in pilot study viz, 10^5^,10^7^ and 10^9^ CFU/ml and bacterial density of LM corresponding to 10^9^ CFU/ml was selected as infectious dose to be used in the main experiment .

### Animals

Healthy albino male mice (*Mus musculus,* 6–8 week old) were purchased from University of Veterinary and Animal Sciences, Lahore and acclimatized in animal house of Department of Zoology, University of the Punjab, Lahore, for one week prior to experimentation. Mice were kept in temperature controlled environment at 28 ± 2 °C with photoperiod 12 h light/dark cycles, humidity 30–35%. They were housed in standard sized cages (12 ″ × 18″ inches) whereas basal diet and fresh water were provided ad libitum.

### Determination of listeria dose (pilot study)

The bacterial density of LM required to induce infection was determined in a pilot study. Mice (*n* = 24) were divided into 4 groups at random, group A, B and C were inoculated once with 200 μl of LM suspension corresponding to cell density of 10^5^, 10^7^ and 10^9^ CFU/ml respectively through intragastric gavage. Group D was maintained as negative control and treated similarly with equal volume of 0.01 M sterile PBS. Re-isolation of LM from visceral organs (spleen, liver and intestine) was used as a criterion of induction of listeriosis. The bacterial density which led to dispersion of LM in liver, spleen and intestine of all animals was used to induce infection in the main study.

### In vivo efficacy and safety of *L. brevis*

A total of 60 mice were divided in four groups I-IV. Groups I and II were used as negative and positive control respectively, group III received only *L. brevis* while group IV received both LB and LM. Mice were fed with *L. brevis* (10^9^ CFU/ml/ mouse) orally by intragastric gavage for 3 consecutive days before oral infection with *L. monocytogene*. Sampling was performed at 3rd, 5th and 7th day of post infection.

### General health scoring (GHS)

Mice were observed daily for clinical signs and symptoms of infection, including animal movement, fur condition, structure of backbone, dehydration and feeding activity and symptoms were scored from 0 to 4. The scores represent following physical attributes (0) Active, alert showing no sign of discomfort; (1) Active, ruffled fur beginning; (2) Slow, ruffled fur with pronounced circumflexed back; (3) Sluggish, ruffled fur, circumflexed back, skin lesion; (4) Sluggish, ruffled fur, circumflexed back, closed and sunken eyes, difficulty in feeding. Animals were scored daily for their health conditions upto 7 days post infection. The GHS of infected animals (II) group were compared with LB treated group (IV).

### Temperature, body weight and feed intake measurements

The body temperature and body weight of each animal was monitored daily throughout the experiment using rectal thermometer and balance (SHIMADZU -ELB300). Similarly, feed intake of each animal was monitored and measured by subtracting feed left behind from the feed given and dividing it by number of the animals in the group.

### *L. monocytogenes* load in different organs

Total 5 mice from each group were sacrificed following ULAM guide lines of euthanization by intra peritoneal injection of ketamine (200 mg/kg of body weight) on 3rd, 5th and 7th day post infection (dpi). Quantification of LM in tissues was performed by removing liver, spleen and intestine aseptically from slaughtered mice. Dilutions of weighed amount of tissue homogenate were prepared in sterile 0.01 M PBS and plated on lithium chloridephenylethanol-moxalactam (LPM) agar for the determination of LM count per gram of the tissue.

### Intestinal bacterial composition

Assessment of intestinal microbial composition of all groups was done by enumerating coliform, Lactic Acid Bacteria (LAB), Total Plate Count (TPC) and Anaerobic Plate Count (APC) on selective media; MacConkey, MRS, nutrient agar under aerobic condition and nutrient agar under anaerobic condition respectively.

### Hematological and serological analysis

Blood was collected by cardiac puncture and kept in EDTA coated and uncoated vails for hematological and serological analysis respectively. For differential leucocytes counting blood smear was prepared on glass slide and fixed with 100% methanol for 5 min. Later on, it was stained with Writght-Giemsa stain for 10 min. Slides were analyzed for lymphocytes, neutrophils, monocytes and eosinophils counts under oil immersion. Serum analysis was performed through fully automated analyzers (Olympus-AU400, USA) using chemical reagents of Beckmann coulter, serum parameters included ALP, ALT/SGPT, AST/SGOT, TBil, Urea, Creatinine, CHOl, TG, HDL and LDL.

### Statistical analysis

Statistical analysis was performed using IBM SPSS software (Statistical Package for Social Sciences) version 21 Chicago, IL, USA. Experiments were performed in triplicate from each sample. All results are presented as group means ± S.E. One Way ANOVA followed by Tukey’s test, independent sample t test and Mann-Whitney U tests were performed as and where appropriate. The *p* value < 0.05 was considered significant.

## Results

The probiotic strain used in the study was Gram positive rod, non-spore former, non-motile and catalase negative. It was identified as *Lactobacillus brevis* on the basis of 16S rRNA sequencing and assigned accession number MF179529 by National Center for Biotechnology Information (NCBI). In pilot study, LM could not be re-isolated from tissues including liver, spleen and intestine of animals exposed to lower bacterial density (10^5^ CFU/ml). Animal exposed to a dose of 10^7^ CFU/ml showed presence of LM in few tissues. It was reisolated from liver and intestine of 2/6 and spleen of 1/6 animals. A single intragastric dose of 10^9^CFU/ml of LM resulted in translocation of pathogen in all tissues of sacrificed animals (Table 1).

**Table 1:** Effect of different doses of LM ATCC19115 on establishment of infection, survival and re-isolation frequency in mice

Parameter	Groups (Dose CFU/ml)			
	A (10^9^)	B (10^7^)	C (10^5^)	D (NIL)
Mortality	0/6	0/6	0/6	0/6
Re-solation				
Liver	6/6	2/6	0/6	0/6
Spleen	6/6	1/6	0/6	0/6
Intestine	6/6	2/6	0/6	0/6

The denominator showed the total number of animals and numerators showed animals exhibiting the respective parameters

In the main study, protective effect of orally administered LB MF179529 (10^9^CFU/ml) against *Listeria* infection (10^9^ CFU/ml) was monitored*.* General health conditions of LB fed group were better than positive control group (*P* ≤ 0.05). The comparison of GSH scores in LB treated and infected group (IV and II) is presented in Table 2.

**Table 2:** Comparison of GHS of LM infected and LB + LM treated group

DPI	Group	General health score					Mann-Whitney U test	
		0	1	2	3	4	Mean rank	*P* value
Day1	Infection	15/15	0/15	0/15	0/15	0/15	0	1.00
	Treated	15/15	0/15	0/15	0/15	0/15	0	
Day2	Infection	0/15	9/15	6/15	0/15	0/15	16.2	.007
	Treated	9/15	6/15	0/15	0/15	0/15	8.2	
Day3	Infection	0/15	3/15	6/15	4/15	2/15	12.5	.005
	Treated	12/15	3/15	0/15	0/15	0/15	4.5	
Day4	Infection	0/10	0/10	3/10	4/10	3/10	10.5	.001
	Treated	10/10	0/10	0/10	0/10	0/10	3	
Day5	Infection	0/10	0/10	2/10	2/10	6/10	10.5	.001
	Treated	10/10	0/10	0/10	0/10	0/10	3	
Day6	Infection	0/5	0/5	1/5	0/5	4/5	8	.001
	Treated	5/5	0/5	0/5	0/5	0/5	2	
Day7	Infection	0/5	0/5	0/5	1/5	4/5	8	.001
	Treated	5/5	0/5	0/5	0/5	0/5	2	

GHS was compared using Mann-Whitney U test among infection and treated groups by mean rank values. Significant (*P* < 0.05) values were observed within 2 groups after 7 days of post infection

**Fig. 1:**
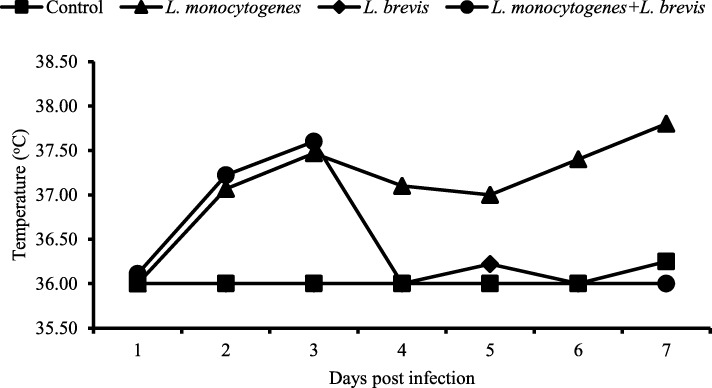
Variation in body temperature among groups. Body temperature of control and *L. brevis* group remain steady throughout the experiment. Body temperature of LB + LM group (IV) increases up to 3 days post infection and then become equal to control group. While body temperature remained high in LM group (II)

Initial body weight among groups was between 30 to 34 g. Among groups there was no significant difference in body weight of animals at the start of experiment. During course of experiment the body weight of negative control (I) and LB fed (III) group remained unaltered, while continuous decrease was observed in positive control (II) group where it reduced from 34 to 28 g. LM + LB treated group (IV) showed initial decrease in body weight at 3rd dpi but later on it increased to level of negative control (Additional file 1: Figure S1). Feed intake of positive control group (II) also decreased in comparison with all other groups (Additional file 2: Figure S2). Body temperature of negative control and LB fed animals (I and III) group remained normal throughout the experiment. Body temperature of LB + LM group (IV) increases up to 3rd dpi and then become equal to negative control (I) group. While body temperature remained high in LM group (II) (Fig. 1).

**Fig. 2:**
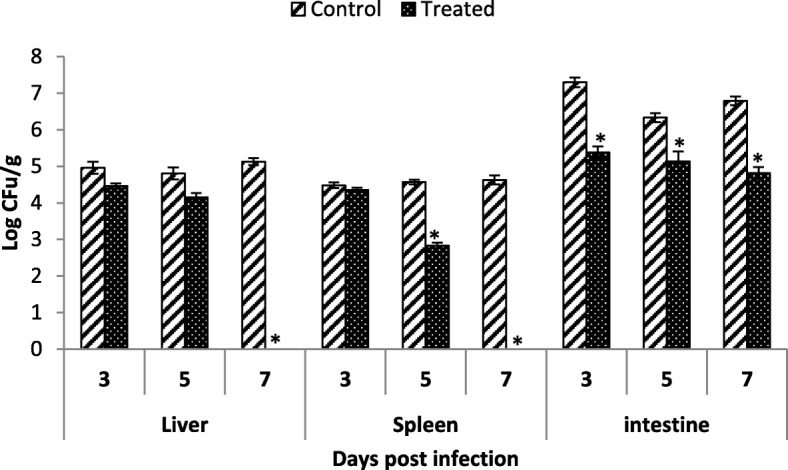
Comparison of orally administered LB on spread of LM in mouse Organs (liver, spleen and intestine). Data was compared using independent sample t-test. Asterics on bar show significant differences at *P* < 0.05

Data on LM translocation in organs revealed that LM load in liver, spleen and intestine was significantly reduced in LB + LM treated (IV) group as compared to positive control (II) group. LM count of group IV was 4.45 CFU/g, 4.15 CFU/g in liver and 4.45 CFU/g and 4.15 CFU/g in spleen at 3rd and 5th dpi respectively. However, it could not be re-isolated at 7th dpi LM from both tissues. On the other hand, its value was 5.3, 5.1 and 4.8 log CFU/g at 3rd, 5th and 7th dpi respectively in intestine. The LM count in group II remained high in all tissues (4.9–5.1 CFU/g in liver, 4.4–4.6 CFU/g in spleen and 6.7–7.3 log CFU/g in intestine) during course of experiment (Fig. 2).

**Fig. 3:**
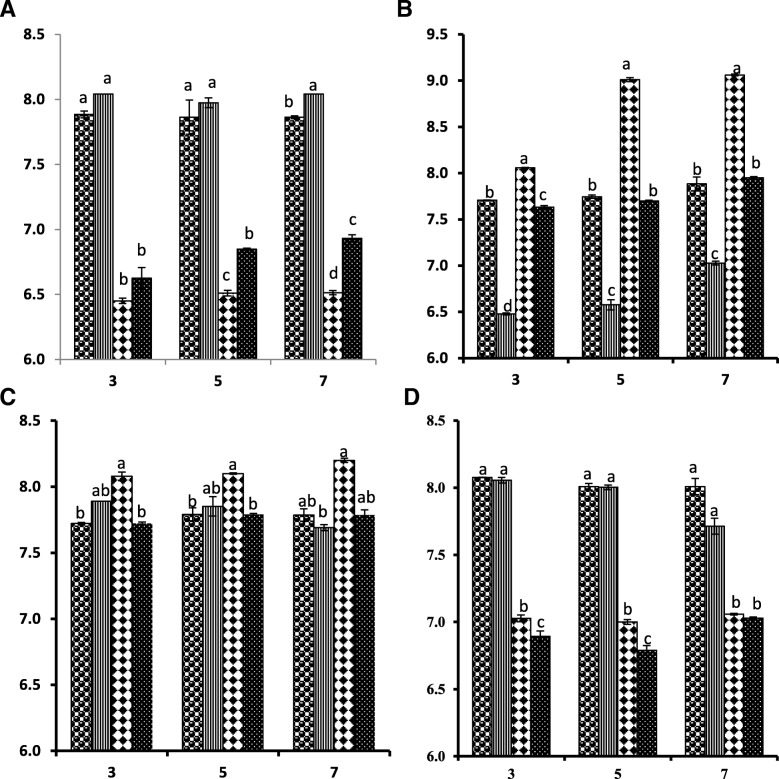
Effect of LM infection and LB treatment on intestinal microbial composition of mice. TPC (Total Plate Count); LAB (Lactic Acid Bacteria); APC (Anaerobic Plate Count) and Coliform count were represented by **a**, **b**, **c** and **d** respectively. Data was analyzed using One way ANOVA followed by DMRT. Bars having no common letters are significantly different from each other at (*P* ≤ 0.05). Error bars represent Standard error of mean. Y-axis presents log CFU/g and X-axis Day post infection (dpi).

Influence of LB treatment on the microbial composition of intestine of mice was analyzed by comparing coliform, LAB, TPC and APC count of group I-IV. LB supplementation lead to significant reduction in TPC and coliform count in group III and IV (Fig. 3a and d). However, significant elevation in LAB count was detected in group III and IV. While infection with LM reduced LAB count in group II when compared with negative control group I (Fig. 3b). The anaerobic plate count (APC) was recorded higher in group III while, in other groups it was similar (Fig. 3c). The pattern of change in TPC, APC, LAB and coliform count was similar at all time points (3rd, 5th and 7th dpi).

Effect of LB treatment on course of infection was further determined by comparing hematological picture of the four groups. A significant decrease in hemoglobin (Hb) and elevation in TLC was noticed at 3rd dpi in positive control (II) group. Platelets count of group II also decreased significantly as compared to negative control group. But LB treated groups (III and IV) showed higher platelets count as compared to negative control group. A significant increase in neutrophils and decrease in lymphocytes was also observed in positive control group (II) while no such alteration was observed in LB treated groups (III and IV). The variation in eosinophils and monocytes levels was not significant among groups (*p* > 0.05) (Table 3). Serological parameters including ALP, ALT/GPT, AST/GOT, TBil, Urea, creatinine, CHOL, TG, HDL and LDL were analyzed during experiment at 3rd, 5th and 7th dpi. No significant change was observed in serological parameters of infected and uninfected groups (data not mentioned).

**Table 3:** Effect of *L.brevis* on the course of listeria infection: changes in hematological parameters

Parameters	dpi	Control	Infection	*L.brevis*	Treated
HB(g/dl)	3	12.7 ± 0.7^a^	10.1 ± 0.2^b^	12.8 ± 0.1^a^	11.6 ± 0.5^ab^
	5	12 ± 0.58^ab^	11.6 ± 0.23^b^	13.37 ± 0.38^a^	12.53 ± 0.20^ab^
	7	12.67 ± 0.67	10.70 ± 0.10	13.20 ± 0.42	11.97 ± 0.55
TLC(X1000)	3	4.75 ± 1.26^b^	7.4 ± 1.0^a^	5.03 ± 2.6^b^	4.9 ± 2.17^b^
	5	4.96 ± 2.60^ab^	5.033 ± 1.33^ab^	5.8 ± 2.08^a^	4.53 ± 3.76^b^
	7	4.70 ± 2.89	5.77 ± 1.33	4.77 ± 145.30	5.33 ± 3.84
Platelets	3	566.3 ± 16.8^b^	378.3 ± 25.5^c^	707.7 ± 36.4^a^	760.0 ± 27.6^a^
	5	565 ± 17^a^	436 ± 34^b^	613 ± 11^a^	661 ± 37^a^
	7	572.00 ± 14.01^a^	472.67 ± 20.73^b^	627.33 ± 15.07^a^	646.33 ± 25.8^a^
Neutrophils %	3	14.3 ± 0.3^b^	26.3 ± 0.7^a^	12.3 ± 1.8^b^	16.0 ± 1.6^b^
	5	14 ± 0.58^b^	22.67 ± 1.76^a^	12.33 ± 1.45^b^	15.33 ± 0.67^b^
	7	13.67 ± 0.88^b^	24.00 ± 2.00^a^	13.33 ± 1.20^b^	13.00 ± 0.58^b^
Lymphocytes %	3	76.7 ± 1.8^bc^	71.0 ± 1.0^c^	85.7 ± 0.9^a^	80.3 ± 1.7^ab^
	5	83 ± 1.53^a^	74.67 ± 2.03^b^	86 ± 1.00^a^	81.33 ± 0.67^a^
	7	80.00 ± 1.15	74.67 ± 2.73	80.00 ± 2.31	81.67 ± 1.45
Monocytes %	3	1.7 ± 0.7	2.0 ± 0.0	2.7 ± 0.3	3.0 ± 0.4
	5	1.33 ± 0.33	2.00 ± 0.00	2.33 ± 0.33	2.00 ± 0.00
	7	2.33 ± 0.33	2.00 ± 0.58	2.00 ± 0.58	2.67 ± 0.33
Eosinophils %	3	1.3 ± 0.3	0.7 ± 0.3	1.0 ± 0.6	0.8 ± 0.3
	5	1.67 ± 0.67	0.67 ± 0.33	1.00 ± 0.00	1.33 ± 0.67
	7	1.33 ± 0.33	1.00 ± 0.58	1.00 ± 0.58	1.33 ± 0.33

Analysis was performed using one-way ANOVA followed by DMRT. Values in rows having no common letter in superscript are significantly different from each other. Data was presented in term of mean ± SE, *P* values < 0.05 were considered significant

## Discussion

*Listeria monocytogenes* is a worldwide public health threat and cause listeriosis by consumption of contaminated food. The current study was performed to comprehend the impact of *Lactobacillus brevis* MF179529 against orally acquired Listeriosis in mice.*Listeria monocytogenes* ATCC 19115 was chosen as a model of bacterial infection in mice. First obstacle in the study was the establishment of LM ATCC 19115 infection in mice. The bacterial density of pathogen for induction of infection varies depending on virulence of strain, route of exposure and dose of pathogen [17]. Since LM is not acid tolerant and most likely to be killed due to low pH in stomach [18]. Higher bacterial density of pathogen is required to induce infection through oral route as compared to intraperitoneal and subcutaneous. Previously, various authors reported successful infection in experimental animals at the dose of 10^9^ of LM [19–21]. Therefore, a pilot study was conducted to determine the infective dose of LM ATCC 19115 using oral transmission route. The oral route was preferred due to its mimicry with human fecal oral route of Listeriosis [22]. Mice were infected with LM ATCC 19115 at three doses (200 μl of 0, 10^5^, 10^7^, 10^9^ CFU/ml) and monitored for morbidity as well as mortality up to 7 dpi. Mice receiving 10^9^CFU/ml displayed clinical signs of infection including circumflex back, ruffled fur and sluggish movement. Additionally, LM was successfully re-isolated from liver, spleen and intestine indicating successful establishment of clinical infections and dissemination of pathogens. On the other hand, in animals exposed to dose of 10^7^ CFU/ml, re-isolation frequency was very low indicating slight infection. In light of these results bacterial density of 10^9^ CFU/ml was decided for main study.

In an effort to analyze the protective effect of local strain *L. brevis* MF179529 against LM ATCC 19115 infection in mice, main study was conducted. Previously, most of the literature reports the inhibitory effects of *Pediococcus acidilactici, Lactobacillus casei*, *Lactobacillus brevis* and *Lactobacillus paracasei* on LM*. L. brevis* MF179529 was one of our lab isolates with significant antimicrobial activity against both Gram positive and Gram negative pathogenic isolates (data not shown). To the best of our knowledge, no data exists on antilisterial effect of LB.

On the basis of previous literature on LAB species, 200 μl of 10^9^ CFU/ml bacterial density of LB MF179529 was offered to mice three days prior to LM infection. [23, 24]. A significant reduction in general health score (GHS) of LB treated animals was noticed throughout. LB treated groups manifested a significant increase in feed intake and gain in body weight as compared to positive control. Likewise, body temperature of LB treated group remained high up to 3 dpi and later on dropped to normal level. These positive effects may be due to limiting growth or dissemination of LM, or change in intestinal microbial equilibrium by LB.

To further strengthen the role of LB in decreasing LM infection, the pathogen load in different organs was quantified. LB treated group displayed low tissue invasion in liver, spleen and intestine relative to positive control group. Interestingly, complete elimination of pathogen was noticed in liver and spleen at 7th dpi. Consistently, LM counts in intestinal tract also reduced gradually from 3rd to 7th dpi, although it was not totaly eliminated. It may be due to secretions of antimicrobial compounds, competition among LB and LM for colonization in GI tract etc. Our findings are consistent with Becattini et al. [25] who reported that commensal gut microbiota play a defensive role against LM infection by preventing its systemic dissemination. Our results are in accordance with previous observations on reduction of LM count among liver, spleen and intestine by pre feeding with *Lactobacillus casei* [26–29].

Of note, another important contribution of LB MF179529 consumption on GI tract was noticed by alteration in intestinal microbial equilibrium. A significant increase in lactic acid bacteria along with reduction of other intestinal bacteria including LM proposed antilisterial activity of LB. It may be due to competitive survival of LB in GI tract [30]. Recovery of high quantity of LAB from the intestine further indicates that LB could successfully withstand the stressed conditions of gastrointestinal tract of mice supporting its probiotic nature. In fact, use of LAB to ablate the intestinal pathogens is pertinent to previous observations on the synergistic effects of antibiotics and corticosteroids in listeriosis [31]. Additionally, antilisterial activity of LB may be an outcome of antimicrobial peptide secretion as reported previously [32].

Hematological studies provide important information about multiple disorders including bacterial infections. Phagocytes especially macrophages and neutrophils plays an important role against bacterial infections. To further confirm the remodeling role of LB, the hematological picture of all groups was compared. Our results indicated that Hb level decreased in infection group making it a high risk factor to the health [33]. It is known since long that LM produces soluble hemolysin which causes lysis of erythrocytes and LM utilize the iron present in hemoglobin to thrive in the host [34]. Absence of anemia in LB treated mice (group III and IV) confirms the antilisterial efficiency of our strain. It may be due to absence of hemolysin production by our strain. Further evidence was provided by TLC, neutrophil and platelet count. Increased TLC and neutrophil count along with decreased platelets counts are indication of establishment and persistence of infection [35, 36]. In LB treated group (III and IV) TLC, neutrophil and platelets counts remained equal to negative control. Similarly, lymphocytes count remained unaltered in all groups except infection group where it decreased significantly. This indicates the clinical importance and non-pathogenic nature of LB.

In vivo safety assessment of LB was performed by comparing hematological and serum biochemical profiles in control and LB fed group. No significant variation in any serological parameter could be recorded indicating safe nature of LB MF179529. In culmination, our results suggest that LB MF179529 from cow intestine is useful for prevention of invasion and facilitates suppression of LM ATCC19115 infection in mice.

## Conclusion

In the present study, our results indicated that oral administration of LB MF179529 provides protective effect to the *Listeria* infected mice. LB reduces LM load in liver, spleen and intestine and favorably perturbates intestinal microbial equilibrium. We suggest that LB MF179529 ingestion has health promoting capacities and a good candidate to be employed as a probiotic against listeria infection. Further studies are needed to best understand the mechanisms by which this microorganism promotes resistance against infectious diseases.

## Additional files

Additional file 1: **Figure S1.** Variations in body weight due to LM infection and its amelioration with LB*.* Body weight at day 1st among all groups statistically similar (*p* ≥ 0.05). (DOCX 19 kb)

Additional file 2: **Figure S2.** Comparison of feed intake among 4 groups days post infection. Comparison was made using One way ANOVA followed by DMRT. Group II mice received only LM and showed significant reduction in feed intake as compared with the control group. In other groups no significant differences in feed intake was observed. Asterics show significant difference at *P* < 0.05. (DOCX 20 kb)

## Abbreviations

APC: Anaerobic Plate Count
dpi: day post infection
GHS: General health scoring
Hb: Hemoglobin
LAB: Lactic Acid Bacteria
LB: *Lactobacillus brevis* MF179529
LM: *Listeria monocytogenes* LM ATCC19115
NCBI: National Center for Biotechnology Information
TPC: Total Plate Count
