# Supplementary material for: Antilisterial efficacy of Lactobacillus brevis MF179529 from cow: an in vivo evidence
Source: BMC Complement Altern Med. 2019 Feb 1;19:37. doi: 10.1186/s12906-019-2444-5 (PMC6359795; doi:10.1186/s12906-019-2444-5)
Supplement: Supplementary file 2 — Figure S2. Comparison of feed intake among 4 groups days post infection. Comparison was made using One way ANOVA followed by DMRT. Group II mice received only LM and showed significant reduction in feed intake as compared with the control group. In other groups no significant differences in feed intake was observed. Asterics show significant difference at P < 0.05. (DOCX 20 kb) [file 12906_2019_2444_MOESM2_ESM.docx]

**Fig. S2.**
